# Hepatitis E Seroprevalence in Europe: A Meta-Analysis

**DOI:** 10.3390/v8080211

**Published:** 2016-08-06

**Authors:** Johannes Hartl, Benjamin Otto, Richie Guy Madden, Glynn Webb, Kathy Louise Woolson, Levente Kriston, Eik Vettorazzi, Ansgar W. Lohse, Harry Richard Dalton, Sven Pischke

**Affiliations:** 1 First Medical Department, University Medical Center Hamburg-Eppendorf, University Hospital Hamburg Eppendorf (UKE), 20246, Hamburg, Germany; b.otte@uke.de (B.O.); alohse@uke.de (A.W.L.); s.pischke@uke.de (S.P.); 2 Royal Cornwall Hospital Trust and European Centre for Environment and Human Health, University of Exeter, Truro TR1 3HD, UK; richie@forfey.com (R.G.M.); glynn.webb@students.pcmd.ac.uk (G.W.); kathy.woolson@nhs.net (K.L.W.); Harry.Dalton@rcht.cornwall.nhs.uk (H.R.D.); 3 Department of Medical Psychology, University Medical Center Hamburg-Eppendorf, 20246, Hamburg, Germany; l.kriston@uke.de; 4 Department of Medical Biometry and Epidemiology, University Medical Center Hamburg-Eppendorf, 20246, Hamburg, Germany; e.vettorazzi@uke.de

**Keywords:** hepatitis E, serosurvey, seroprevalence, Europe, developing countries, genotype 3, assay, anti-HEV IgG

## Abstract

There have been large numbers of studies on anti-HEV IgG seroprevalence in Europe, however, the results of these studies have produced high variability of seroprevalence rates, making interpretation increasingly problematic. Therefore, the aim of this study was to develop a clearer understanding of anti-HEV IgG seroprevalence in Europe and identify risk groups for HEV exposure by a meta-analysis of published studies. **Methods:** All European HEV-seroprevalence studies from 2003 to 2015 were reviewed. Data were stratified by assay, geographical location, and patient cohort (general population, patients with HIV, solid-organ transplant recipients, chronic liver disease patients, and individuals in contact with swine/wild animals). Data were pooled using a mixed-effects model. **Results:** Four hundred thirty-two studies were initially identified, of which 73 studies were included in the analysis. Seroprevalence estimates ranged from 0.6% to 52.5%, increased with age, but were unrelated to gender. General population seroprevalence varied depending on assays: Wantai (WT): 17%, Mikrogen (MG): 10%, MP-diagnostics (MP): 7%, DiaPro: 4%, Abbott 2%. The WT assay reported significantly higher seroprevalence rates across all cohorts (*p* < 0.001). Individuals in contact with swine/wild animals had significantly higher seroprevalence rates than the general population, irrespective of assay (*p* < 0.0001). There was no difference between any other cohorts. The highest seroprevalence was observed in France (WT: 32%, MP: 16%) the lowest in Italy (WT: 7.5%, MP 0.9%). Seroprevalence varied between and within countries. The observed heterogeneity was attributed to geographical region (23%), assay employed (23%) and study cohort (7%). **Conclusion**: Seroprevalcence rates primarily depend on the seroassy that is used, followed by the geographical region and study cohort. Seroprevalence is higher in individuals exposed to swine and/or wild animals, and increases with age.

## 1. Introduction

Hepatitis E virus (HEV) is hyperendemic in many developing countries, especially in Southeast Asia and Africa, where it causes acute hepatitis predominantly in young adults. Hepatitis E is usually an acute self-limiting illness, except in pregnant women and patients with underlying chronic liver disease, who have mortality rates of up to 25% and 70%, respectively [1]. The disease is caused by HEV genotypes 1 and 2, which are obligate human pathogens spread orofaecally via contaminated water supplies. Cases occur both sporadically and occasionally in large outbreaks. Every year, an estimated 20 million HEV infections occur resulting in more than three million clinical cases and 70,000 deaths [2].

In developed regions, hepatitis E was previously thought to be rare and largely restricted to travelers returning from endemic developing nations. This notion was mistaken [3]. Data published throughout the last ten years show quite clearly that locally-acquired hepatitis E in Europe and other developed countries is common. In contrast to imported HEV genotype 1 or 2 infections, autochthonous hepatitis E in Europe and most developed countries is caused by genotype 3 and features different clinical characteristics, in particular, it is largely a porcine zoonosis [4]. In addition, chronic HEV infection occurs in the immunosuppressed. This includes transplant recipients receiving immunosuppressive therapy [5,6,7], patients with haematological malignancy [8], and individuals infected with HIV and low T CD4 count [9]. While patients with underlying chronic liver disease quite frequently develop acute/subacute liver failure, with a mortality of 27% [10], hepatitis E in developed countries is mostly asymptomatic [11,12]. As a result, HEV has found its way into the human blood supply, with a surprisingly high frequency of viremic donors in some European countries [13,14,15,16].

One approach which helps our understanding of past and present infections in a community, is the study of IgG seroprevalence. There have been a large number of anti-HEV IgG seroprevalence studies in Europe that have been published over the last few years. The results of these studies have been difficult to interpret, as seroprevalence estimates in Europe range from 0.6% to 52.5% [17,18] and the wide range of results reported appear to depend on a number of variables. In particular, there seems to be a large variability in seroprevalence estimates depending on the used anti-HEV IgG assay. The aim of this study was to develop a clearer understanding of anti-HEV IgG seroprevalence in Europe by a meta-analysis of published studies. In order to produce a realistic overview of anti-HEV seroprevalence, we performed a detailed calculation of European anti-HEV-seroprevalence rates depending on the used seroassay, the study cohort, and geographic location.

## 2. Methods

### 2.1. Search Strategy and Selection Criteria

**Figure 1:**
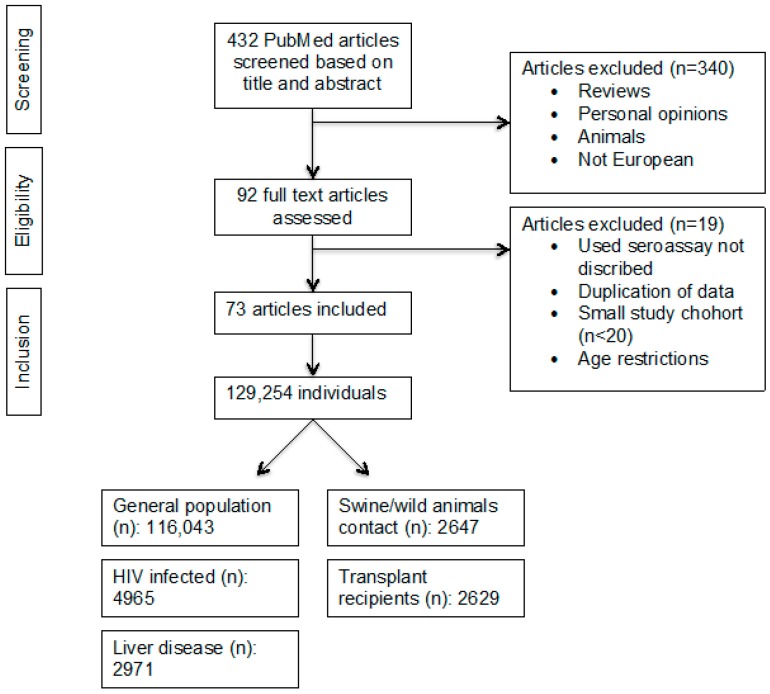
Search algorithm for the anti-HEV IgG seroprevalence meta-analysis.

The meta-analysis is reported in line with the guidelines of the “Preferred Reporting Items for Systematic Reviews and Meta-Analyses” (PRISMA) [19,20]. A keyword literature search was performed in PubMed using the terms “hepatitis E seroprevalence”, “hepatitis E serosurvey”, “hepatitis E epidemiology”, combined with “country”, restricting the search to publication dates between January 2003 and May 2015. Studies that did not report the type of assay employed, reported seroprevalence rates in children, or had a sample size fewer than 20 were not included. No language restrictions were applied. Only studies regarding seroprevalence rates in countries entirely contained in continental Europe were included (i.e., excluding Turkey and the Russian Federation). Original abstracts were obtained and assessed in detail for inclusion. Following abstract review, the full papers of the included studies were reviewed (Figure 1). Data were reviewed independently by two investigators (J.H. and S.P.) and any disagreements were resolved by discussion.

### 2.2. Data Extraction

The following information was extracted from each study: first author, journal, year of publication, country, diagnostic assay used, number of patients, seroprevalence, type of patient cohort, age, and gender of subjects. Data were stratified for three variables: assay employed, country of study, and nature of study-cohort. We focused on five different study-cohorts: general population/blood donors, patients infected with HIV, solid organ transplant recipients, patients with chronic liver disease, and individuals with contact with swine/wild animals. The latter included veterinarians, farmers, forestry workers, and slaughterhouse workers (Figure 1). Some studies provided information for more than one study category (e.g., a different assay was employed for one or more study cohorts), so the total number of data points exceeded the number of studies. Supplementary Table S1 shows the number of included studies per country, while supplementary Table S2 provides detailed information on all included studies and data extracted from each study.

### 2.3. Study Quality

Primary study quality criteria which were applied were: sample size ≥ 20; identification of assay employed and confirmation that it was used according to the manufacturer’s instructions; absence of age restriction of the study cohort (e.g., study limited to only age > 65 years, children and/or adolescents < 18 years were excluded); population based study. Studies that did not meet all of the study quality criteria were excluded from the meta-analysis.

### 2.4. Statistical Analysis

We estimated prevalence of anti-HEV IgG by pooling the study data in order to run a meta-analysis. We used a mixed-effects model including the assay employed, the country of study and study cohort information as single or interacting moderators. We used the double arcsine transformation method for variance stabilization [21] and a restricted maximum likelihood (REML) estimator for prevalence estimation. The analysis was conducted using the R statistical platform (version 3.1.2) and The metafor Package (version 1.9-5) [22]. The I2 statistic was used to estimate the amount of heterogeneity accounted for by each model [23].

## 3. Results

The PubMed search identified 432 publications, which were screened by title and abstract. Ninety-two articles were considered for full text assessment and reviewed by two independent reviewers. Seventy-three studies from 11 countries were included in the final data analysis, with reported seroprevalence rates ranging from 0.6% to 52.5% [17,18]. These studies were composed of a total of 129,254 individuals who were tested for anti-HEV IgG and included 116,043 individuals from the “general population” (healthy individuals), 4964 infected with HIV, 2629 solid organ transplant recipients, 2971 patients with chronic liver disease, and 2647 with contact with swine/wild animals. The study flowchart is shown in Figure 1.

### 3.1. Anti-HEV IgG Assays Employed

**Figure 2:**
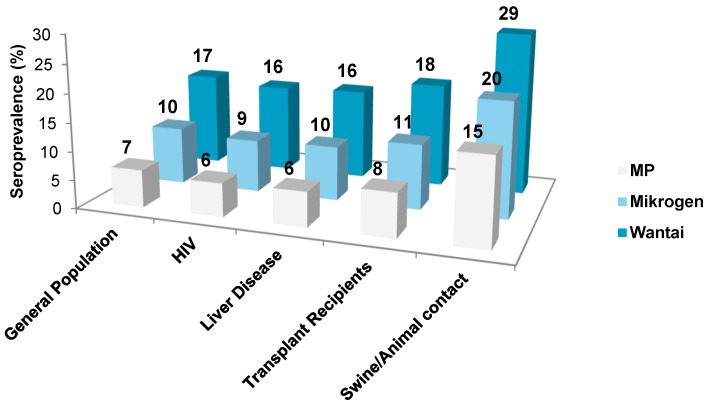
The relationship between anti-HEV IgG seroprevalence rates and the assay employed in different study cohorts. The difference between Wantai (WT) vs. Mikrogen (MG) and WT vs. MP was statistically significant after adjusting for study cohort (WT vs. MG: *p* < 0.05; WT vs. MP: *p* < 0.001).

The pooled anti-HEV IgG seroprevalence rates determined by different commercial assays showed large variability with reported seroprevalence rates ranging from 2% to 17% (Table 1). The most frequently used assays were Wantai (WT), Mikrogen (MG), and MP-diagnostics (MP). For these three assays the pooled seroprevalence rates for the general population were: WT 17% (95% confidence interval, 12.2%–21.2%), MG 10% (3.2%–20.4%), and MP 7% (1.9%–14.1%). For the general population the WT assays reported significantly higher seroprevalence rates compared to MG (*p* < 0.05) and MP (*p* < 0.01). This pattern was observed in all study cohorts (Figure 2). Nine studies assessed seroprevalence rates in a given population by using different assays and found a large discordance between determined seroprevalence rates (Table 2). Importantly, estimated seroprevalence rates in these studies showed the same pattern as seen in Figure 2, with the highest seroprevalence estimated by WT, followed by MG and MP.

### 3.2. Study Cohort

**Figure 3:**
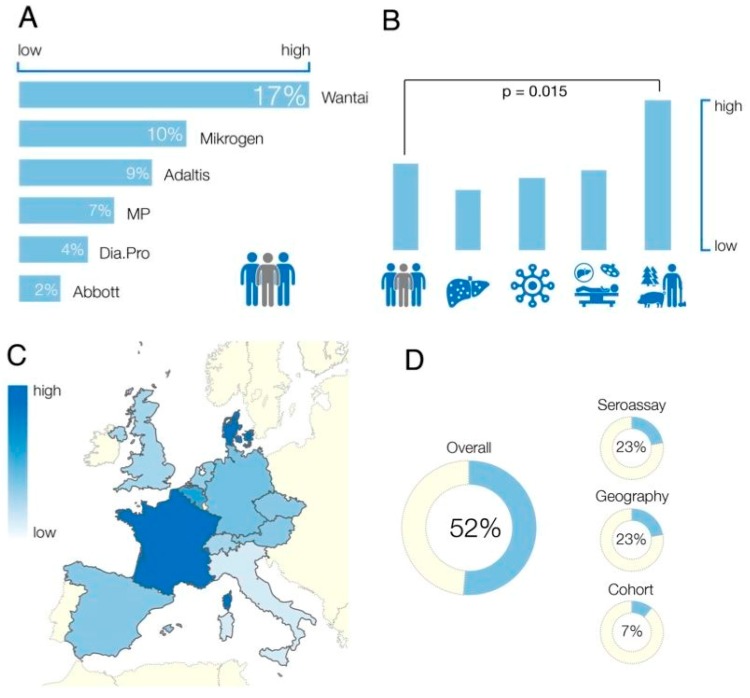
(**A**) Anti-HEV IgG seroprevalence rates in the general population dependent on the used seroassay; (**B**) comparison of estimated seroprevalence rates adjusted for patient cohort. Patient cohorts from left to right: general population, liver diseases, HIV infections, transplant recipients, swine/wild animal contact (farmers, veterinarians, slaughterhouse workers, forestry workers); (**C**) calculated anti-HEV seroprevalence in different European countries. Exact seroprevalence rates are displayed in Table 3; and (**D**) amount of heterogeneity explained by used seroassay, study cohort, and geographical location.

Individuals with close contact to swine/wild animals had higher seroprevalence rates compared to the other study cohorts (Figure 2 and Figure 3B). This finding was independent of the assay used. Compared to the general population, individuals exposed to swine/wild animals had seroprevalence rates of 17% (12.2%–21.2%) vs. 28% (16.9%–41.7%) (*p* < 000.1) using WT, 10% [3.2%–20.4%] vs. 20% (5.5%–40.9%) (*p* < 0.001) using MG, and 7% (1.7%–14.1%) vs. 15% (3.6%–33%) (*p* < 0.001) when the MP assay was employed. We found no statistically significant difference between the seroprevalence rates between any of the other study cohorts (Figure 3B).

### 3.3. Geographical Location

Studies included in the meta-analysis were from Germany (n = 15), France (n = 14), Spain (n = 11), United Kingdom (n = 9), the Netherlands (n = 8), Italy (n = 6), Denmark (n = 3), Switzerland (n = 3), Austria (n = 2), Belgium (n = 1), and the Czech Republic (n = 1). Independent of the assay employed, there were large differences in calculated seroprevalence rates between countries (Figure 3C). In the general population, the highest seroprevalence was estimated for France (WT 32% [95% confidence interval 19–47] %; MP 16 [4–35] %) and the lowest for Italy (WT 8 [1–21] %; MP 1 [0–12] %) followed by the UK (WT 13 [10–17] %; MP 3 [0–10] %). The calculated seroprevalence in France was significantly higher than in the UK (*p* < 0.001) and Italy (*p* < 0.001; Figure 3C). The difference between the UK and Italy was not significant (*p* > 0.05). 

In addition to differences in seroprevalence between countries, differences in seroprevalence have also been reported within countries. Such regional differences have best been described in France and the UK. The highest anti-HEV seroprevalence rates (52.5%) throughout Europe were reported in the Midi-Pyrénées region of Southwest France [18]. Although the Midi-Pyrénées region is relatively small (45,348 km^2^, population 2,926,592), the anti-HEV IgG seroprevalence rate varies significantly within different administrative areas within the region. For example, in a study employing the WT assay, the seroprevalence in the Ariège area (4890 km^2^, population 152,366) was 71% compared to 23.2% in Aveyron and Lot (13,952 km^2^, population 450,575) [24].

In general, a significantly higher seroprevalence was reported for the south when compared to the north of France [25]. However, this difference failed to reach statistical significance when we compared the pooled anti-HEV seroprevalence from regions in Southern France (Toulouse, Marseille, Rhones-Alpes, regions of Languedoc-Roussillon, Rhone-Alpes, Midi-Pyrénées, Hyere) with the rest of the country (*p* > 0.05).

A north/south gradient has also been described in the UK. The lowest seroprevalence in central Europe was found in Scotland (WT 4%) [26] with a calculated seroprevalence of 13 [10–17] % (WT) in the rest of the UK (north vs. south: *p* < 0.05).

### 3.4. Age and Gender

Forty-six of the 73 studies provided data on age. Most studies (n = 33) only provided the median age of participants. Analysis of age was further complicated by the fact that studies evaluating whether anti-HEV IgG seropositivity increases with age used age categories that were not consistent between studies. Therefore, no detailed statistical analysis of the influence of age on anti-HEV seroprevalence could be performed. However, of the 18 studies which were evaluable in terms of age all but one [27] showed that anti-HEV seroprevalence increases with age. Forty-five of the 73 studies provided information on sex. No significant difference in HEV seroprevalence was found between genders.

### 3.5. Study Heterogeneity

Twenty-three percent of the observed heterogeneity in anti-HEV seroprevalence studies was due to the assay employed and the geographical region, respectively. The study cohort accounted for an additional 7% of the heterogeneity (Figure 3D). Thus, the majority of the observed heterogeneity (52%) could be attributed to these three factors.

## 4. Discussion

The present study is the first meta-analysis of reported anti-HEV IgG seroprevalence rates in Europe. Seventy-three articles compromising a total of 129,254 individuals were included. Published seroprevalence rates ranged from 0.6% to 52.5%. Considerable heterogeneity was found between studies that was mainly attributable to the anti-HEV IgG assay employed, the geographical location, and the type of study cohort. Therefore, we calculated anti-HEV IgG seroprevalence depending on geographical region, used seroassay, and studied population, which has, in our opinion, produced a realistic overview of HEV seroprevalence in Europe and may allow a better interpretation of previous and future studies on HEV seroprevalence in developing countries.

The majority of studies demonstrated a high exposure of the European population to HEV, which contrasts with the relatively low (but increasing) number of reported hepatitis E cases in Europe. This suggests that the majority of infections are asymptomatic and/or unrecognised [11,12]. Recently it has been shown that locally-acquired infections with HEV genotype 3 tend to take a less severe course than imported HEV infections [28].

The anti-HEV IgG assay employed had a significant influence on the reported seroprevalence rates. Commercial assays vary considerably in their performance with a large range of sensitivities and specificities [29,30]. Nine studies assessed seroprevalence rates in a given population using different assays (Table 2), which resulted in very different seroprevalence rates depending on the employed assay. For instance, Schnegg et al. found a seroprevalence of 4.9% in Swiss blood donors by using the MP assay, while the seroprevalence in the same population was more than 5 times higher (21%) when determined by the WT assay [31]. Compared to all the other assays, the anti-HEV IgG WT assay, in general, produces higher seroprevalence estimates and has been regarded by many as the “gold standard” in the field [32]. The reason for this is that it has a validated sensitivity for detecting distant infection of 98% [33]. None of the other assays have been validated in this way and at least one other commonly used assay has a sensitivity for detecting distant infection of < 50% [33]. However, the WT assay is regarded by some observers as producing seroprevalence results that are so high that they stretch biological plausibility and are simply a reflection of the assay’s poor specificity. In common with all other commercial assays, the WT assay has not been fully assessed in terms of specificity for distant infection. However, there are adult populations that have very low seroprevalence when the WT assay is used. These include Fiji (2%) [34], New Zealand (4%) [35], and Scotland (4.6%) [26] and, in the hyperendemic area of Southwest France, the seroprevalence is 2% in children aged 2–4 years [18]. Taken together, these data suggest that the WT IgG assay does not lack specificity in determining previous infection. Furthermore, several studies have reported that a considerable number of blood donors have HEV RNA at time of donations (Table 4), which lends weight to the notion that the WT assay gives credible estimates of viral pressure on populations over time.

**Figure 4:**
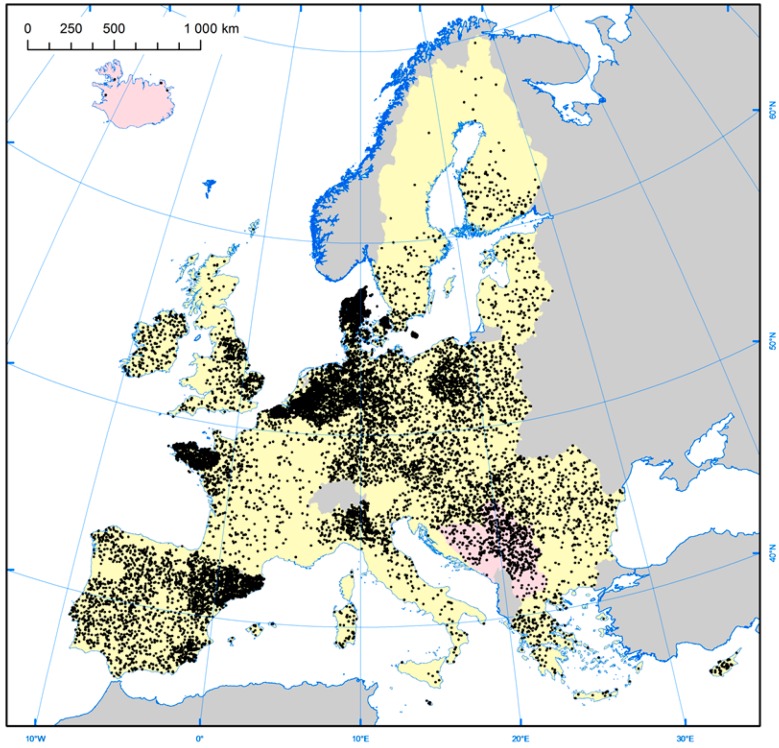
A diagrammatic representation of the distribution of the number of sows in the European Union [44].

The current study suggests that, after accounting for differences in the employed assays anti-HEV seroprevalence varies between countries, the highest anti-HEV IgG seroprevalence rates are found in France and the lowest in Italy. An important route of infection for HEV is thought to be via the food chain, due to consumption of HEV-infected pork products. Another possible route of infection is due to environmental contamination with HEV. Interestingly, while France has the highest seroprevalence, the country’s consumption of pork is less than in many other European countries (Table 5), as is the number of pigs produced (Table 5 and Figure 4). It would appear that there is no clear relationship, at least at the country level, between anti-HEV seroprevalence and either pork consumption or production (Table 5). However, anti-HEV IgG seroprevalence may relate to differences in culinary practices between countries/regions and/or different rates of viremia/viral loads of HEV-contaminated pork products in the human food chain. In France, for example, HEV has been found to contaminate a range of pork-based food delicacies, often at very high viral loads [41]. This includes figatellu, which is a pork liver sausage commonly consumed in Southern France. Figatellu is meant to be consumed after cooking, but is commonly eaten raw and has been implicated in a number of cases of locally-acquired hepatitis E in Southern France [18,42].

Anti-HEV IgG seroprevalence not only varies between countries, but also within countries, with significant variance between regional areas. The finding that there is more than a three-fold difference in seroprevalence between the area of Ariège (71%) and Aveyron (23.2%), which are both in Southern France and less than 200 km apart, is of particular interest [24]. Such a huge difference in seroprevalence is unlikely to be solely explained by differences in culinary culture in inhabitants of neighbouring areas of Southern France, and suggest that unknown environmental factors may have a role as the source of human infection. This hypothesis is lent further weight by the recent finding that in Cornwall, UK over 50% of patients presenting with hepatitis E lived within 2 km from the coast [47].

Despite the heterogeneity between studies, two themes have emerged: HEV seroprevalence is higher in individuals exposed to swine/wild animals and increases with age. The observation that anti-HEV seroprevalence is higher in individuals exposed to swine and wild animals further supports the notion. Contrary to the widely held view that most infections in Europe are caused by consumption of pork products contaminated with HEV [48], environmental factors may play an important role in human infection. The finding that anti-HEV IgG seroprevalence increases with age comes as no surprise and is likely to reflect cumulative life-time exposure to HEV, which appears to be similar in men and women.

Nevertheless, the finding that no significant difference in HEV seroprevalence was observed between genders is somewhat surprising, since symptomatic HEV infection in developed countries is much more common in middle-aged and elderly men [4,34]. Given that HEV exposure seems to be unrelated to sex, we assume that host factors must explain why these populations are more likely to develop overt hepatitis.

We did not specifically study changes of seroprevalence over time due to the very limited number of existing studies. It appears that, at least in the UK, Denmark, and Germany, seroprevalence rates have fallen compared to 20 years ago [26,49,50,51]. There appears to be a cohort effect, related to date of birth, suggesting that high seroprevalence rates seen in the elderly are due to a surge of infection in the 1950s and 1960s. However, a study from the Netherlands has shown a recent dramatic increase in seroprevalence in young adults [52]. This has been accompanied by an increase in viremic blood donors from 1:2761 in 2011–2012 [37] to 1:600 in 2014 [14]. These data should be kept in mind when interpreting Figure 3C, which displays the geographical variation in seroprevalence since, for unknown reasons, HEV infection may have become hyperendemic in the Netherlands in recent years.

We chose to exclude seroprevalence studies prior to 2003 as we felt most were fundamentally flawed. The reason for this is that such studies largely used “first generation” anti-HEV IgG assays and produced seroprevalence estimates of < 5% due to very poor assay sensitivity, which previously led us to the erroneous notion that HEV in Europe was uncommon and almost universally imported from endemic developing countries [3].

Looking ahead, seroprevalence studies should consider seroprevalence at regional as well as national level and attempt to minimize heterogeneity. This means that study cohorts need to be uniformly delineated and the performance of assays employed at detecting distant infection need to be accurately defined. For example, we currently do not know much about assay performance at detecting distant infection in some important study cohorts such as immunosuppressed transplant recipients, individuals with HIV, and patients with chronic liver disease. To this end studies are currently in progress to determine the sensitivity and specificity of existing anti-HEV IgG assays at detecting distant infection, together with a project to establish WHO reference materials for serological assay development. The results are awaited with interest.

In conclusion, anti-HEV IgG seroprevalence rates in Europe ranged from 0.6% to 52.5%. Considerable heterogeneity was found between studies, mainly attributable to the assay employed, the geographical location and the type of study cohort. HEV seroprevalence varies both between and within countries, is higher in individuals exposed to swine/wild animals and increases with age. Future seroprevalence studies should minimize heterogeneity by defining assay performance and study cohorts more accurately.

## Figures and Tables

**Table 1:** Anti-HEV IgG seroprevalence rates determined by different commercial assays for different study cohorts.

Study cohort	Wantai	Mikrogen	MP	Abbott	Adaltis	Dia.Pro	Others
General Population (%)	16.90	10.11	6.50	2.29	8.72	4.35	12.48
Sample size (n)	88,204	1777	14,385	1077	nd *	5,176	3667
Liver Disease (%)	16.05	9.55	6.13	2.02	8.2	3.94	11.86
Sample size (n)	nd *	41	801	129	nd *	nd *	2000
Transplant recipients (%)	18.36	11.42	7.69	2.97	9.96	5.22	13.91
Sample size (n)	415	124	1328	262	64	448	52
HIV (%)	15.69	9.26	5.900	1.88	7.93	3.75	11.55
Sample size (n)	2047	nd *	1579	123	429	548	238
Swine/Animal Contatct (%)	28.51	20.13	15.26	8.37	18.25	11.82	23.21
Sample size (n)	101	709	1354	202	43	nd *	995

* For combinations of seroassays and study cohorts for which reported seroprevalence rates were not determined (nd), the seroprevalence was calculated by using a restricted maximum likelihood estimator model (R statistical platform and The metafor Package).

**Table 2:** Studies assessing anti-HEV IgG seroprevalence in a given population by different anti HEV‑IgG assays (n = 9).

Journal	Year	First Author	Cohort Size	Sero-Prevalence	Assay	Cohort	Country
**Transfusion**	2015	Holm	504	10.7	Other	GP	Denmark
**Transfusion**	2015	Holm	504	19.8	Wantai	GP	Denmark
**J Clin Virol**	2013	Rossi-Tamisier	64	10.9	Adaltis	Tx	France
**J Clin Virol**	2013	Rossi-Tamisier	64	31.3	Wantai	Tx	France
**J Infect Dis**	2012	Wenzel	200	18	Mikrogen	GP	Germany
**J Infect Dis**	2012	Wenzel	200	4.5	MP	GP	Germany
**J Infect Dis**	2012	Wenzel	200	29.5	Other	GP	Germany
**Hepatology**	2014	Wenzel	1092	14.5	Mikrogen	GP	Germany
**Hepatology**	2014	Wenzel	1092	34	Other	GP	Germany
**Med Mibrobiol Immun**	2014	Krumbholz	235	8.5	Mikrogen	GP	Germany
**Med Mibrobiol Immun**	2014	Krumbholz	235	2.6	MP	GP	Germany
**Med Mibrobiol Immun**	2014	Krumbholz	235	7.7	Other	GP	Germany
**Med Mibrobiol Immun**	2014	Krumbholz	302	17.9	Mikrogen	SW	Germany
**Med Mibrobiol Immun**	2014	Krumbholz	302	3.5	MP	SW	Germany
**Med Mibrobiol Immun**	2014	Krumbholz	302	13.2	Other	SW	Germany
**Epidemiol Infect**	2008	Bouwknegt	644	1.7	Abbott	GP	Netherlands
**Epidemiol Infect**	2008	Bouwknegt	644	4.2	MP	GP	Netherlands
**Epidemiol Infect**	2008	Bouwknegt	49	8.1	Abbott	SW	Netherlands
**Epidemiol Infect**	2008	Bouwknegt	49	12.2	MP	SW	Netherlands
**Epidemiol Infect**	2008	Bouwknegt	153	5.2	Abbott	SW	Netherlands
**Epidemiol Infect**	2008	Bouwknegt	153	3.9	MP	SW	Netherlands
**Transfusion**	2014	Sauleda	10,000	10.72	Mikrogen	GP	Spain
**Transfusion**	2014	Sauleda	10,000	19.96	Wantai	GP	Spain
**PLoS One**	2013	Schnegg	550	4.9	MP	GP	Switzerland
**PLoS One**	2013	Schnegg	550	4.2	Dia.Pro	GP	Switzerland
**PLoS One**	2013	Schnegg	550	21.2	Wantai	GP	Switzerland
**J Med Virol**	2010	Bendall	500	3.6	MP	GP	UK
**J Med Virol**	2010	Bendall	500	16.2	Wantai	GP	UK

GP: general population; Tx: transplant recipients; SW: swine/animal contact.

**Table 3:** Calculated seroprevalence rates for the general population.

Title	Abbott	Adaltis	Dia.Pro	Mikrogen	MP	Other	Wantai
Austria	1.9% *	0.7% *	6.6% *	8.9% *	3.9% *	9.3% *	13.9%
Belgium	4.5% *	2.5% *	10.9% *	13.8% *	7.4% *	14.3%	19.7% *
Czech Republic	1.5% *	0.5% *	5.9%	8.1% *	3.3% *	8.5% *	12.9% *
Denmark	4.8% *	2.8% *	11.4% *	14.3% *	7.8% *	15.2%	19.8%
France	12.0% *	8.7%	21.1% *	24.7% *	16.3%	25.4%*	31.9%
Germany	2.6%	1.1% *	7.8% *	10.3%	4.8%	10.8%	29.5%
Italy	0.1% *	0.1% *	2.4%	3.9% *	0.9% *	4.1%	7.5%*
Netherlands	1.8%	0.6% *	6.4%	8.7% *	3.7%	9.1%	27.0%
Spain	2.2%	0.9% *	7.1%	9.5% *	4.3%	10.0%*	14.7%
Switzerland	1.8% *	0.6%*	4.2%	8.8%	4.2%	9.2%	21,2%
UK	1.4% *	0.4% *	5.7% *	7.9% *	3.2%	8.3% *	12.7%

* For combinations of seroassays and countries for which reported seroprevalence rates were not determined, the seroprevalence was calculated using a restricted maximum likelihood estimator model (R statistical platform and The metafor Package).

**Table 4:** HEV viremia and seroprevalence in blood donors in European countries.

Country	Blood Donors HEV RNA Positive	HEV IgG Seroprevalence	Reference
Midi-Pyrénées, Southwest France *	1:1438 (1:2200) **		Gallian et al., 2014 [15]
		52.5%	Mansuy et al., 2011 [18]
**Germany**	1:1200		Vollmer et al., 2012 [16]
	1:4525		Baylis et al., 2012 [36]
		29.5%	Wenzel et al., 2013 [29]
**The Netherlands**	1:2671	27.0%	Slot et al., 2013 [37]
**England**	1:2848	*	Hewitt et al., 2014 [13]
	1:7000	12.0%	Ijaz et al., 2012 [38]
		16.0%	Beale et al., 2011 [39]
		16.0%	Dalton et al., 2008 [4]
**Sweden**	1:7986	NA	Baylis et al., 2012 [36]
**Austria**	1:8416	13.5%	Fischer et al., 2015 [40]
**Scotland**	1:14,520	4.7%	Cleland et al., 2013 [35]

Seroprevalence studies have been restricted to those employing the highly-sensitive and partially-validated Wantai anti-HEV IgG assay. HEV RNA was genotype 3 in all cases. * deconstructed solvent‑detergent treated mini-pools. NA: not available. ** Midi-Pyrénées/Méditerranées: 1:1438, France: 1:2200.

**Table 5:** HEV seroprevalence and pig production/consumption.

Country	Estimated Human HEV Seroprevalence (WT Assay)	Number of Pigs Slaughtered 2013 (Millions)	Human Population 2013 (Millions)	Pigs/Human Ratio **	Pork Consumption (Thousand Tons) ***	Pork Consumption (Kg) per Capita ***
**France**	31.9	23,747	63.9	0.37	1931	30.2
**Germany**	29.5	58,628	80.6	0.72	4358	54.1
**Denmark**	19.8	19,108	5.6	3.41	352	62.9
**Netherlands**	27.0	14,014	16.8	0.83	640	38.1
**Belgium**	19.7	11,915	11.2	1.06	452	40.4
**Spain**	14.7	41,418	46.6	0.31	2363	50.7
**Switzerland**	13.8	No comparative data	8.1	NA	201 *	24.8
**Austria**	13.9	5417	8.5	0.64	474	55.8
**Czech Republic**	12.9	2652	10.5	0.25	437	41.6
**UK**	12.7	10,299	64.1	0.16	1542	24.1
**Italy**	7.5	13,099	59.8	0.22	2451	41.0

NA = not available; * 2010 data: [43,44,45,46]; ** Product-moment correlation between HEV seroprevalence and pig human/ratio r = 0.197 (p = 0.630); *** Product-moment correlation between HEV seroprevalence and pork consumption r = −0.195 (p = 0.591).

## Supplementary Materials

The following are available online at www.mdpi.com/1999-4915/8/8/211/s1, **Table S1.** Number of included studies per country; **Table S2.** All included studies and data extracted from each study.
