# Supplementary material for: Hepatitis E Seroprevalence in Europe: A Meta-Analysis
Source: Viruses. 2016 Aug 6;8(8):211. doi: 10.3390/v8080211 (PMC4997573; doi:10.3390/v8080211)
Supplement: Supplementary file 1 [file viruses-08-00211-s001.pdf]

Supplementary Materials: Hepatitis E Seroprevalence in Europe: A Meta-Analysis

Johannes Hartl, Benjamin Otto, Richie Guy Madden, Glynn Webb, Kathy Louise Woolson, Levente Kriston, Eik Vettorazzi, Ansgar W. Lohse, Harry Richard Dalton, Sven Pischke

Table S1. Number of included studies per country

Country	Number of Studies per Country
Austria	2
Belgium	1
Czech Republic	1
Denmark	3
France	14
Germany	15
Italy	6
Netherlands	8
Spain	11
Switzerland	3
UK	9
**TOTAL**	73

Table S2. All included studies and data extracted from each study

Journal	Year	First Author	Subcohort	Number of Patients	Sero-Prevalence	Test	Cohort	Country
PloS One	2014	Lagler		997	14.3	Wantai	General Population	Austria
PLoS One	2015	Fischer		58,915	13.55	Wantai	General Population	Austria
Acta Gastroenterol Belg	2012	Van Hoecke F		100	14	Other	General Population	Belgium
Epidemiol Mikrobiol Imunol	2014	Strakov		230	5.7	Dia.Pro	General Population	Czech Republik
Clin infect dis	2008	Christensen	samples collected in 1983	169	32.5	Other	General Population	Denmark
Clin infect dis	2008	Christensen	samples collected in 1983	291	50.3	Other	Swine/Forestry Workers	Denmark
Clin infect dis	2008	Christensen	samples collected 2003	461	20.6	Other	General Population	Denmark
**Tansfusion**	**2015**	**Holm**		**504**	**10.7**	**Other**	**General Population**	**Denmark**
**Tansfusion**	**2015**	**Holm**		**504**	**19.8**	**Wantai**	**General Population**	**Denmark**
AIDS	2010	Renou	southern patients	112	3	Adaltis	Immune Deficiency	France
AIDS	2010	Renou	northern patients	133	9	Adaltis	Immune Deficiency	France
Emerg Infect Dis	2011	Mansuy		512	52.5	Wantai	General Population	France
Eur J Clin Microbiol Infect Dis	2014	Buffaz		142	29	Wantai	General Population	France
Int J Infect Dis	2013	Vernier		43	48.8	Wantai	General Population	France
Int J Infect Dis	2013	Vernier		43	30.2	Adaltis	Swine/Forestry Workers	France
J Clin Microbiol	2007	Boutrouille		1998	3.2	MP	General Population	France
J Clin Virol	2012	Carpentier		593	31	MP	Swine/Forestry Workers	France

J Clin Virol	2012	Maylin	261	1.5	MP	Immune Deficiency	France
J Clin Virol	2012	Maylin	46	6.5	MP	Transplant	France
J Clin Virol	2013	Chaussade	322	26.2	MP	General Population	France
J Clin Virol	2013	Chaussade	231	36	MP	Immune Deficiency	France
J Clin Virol	2013	Chaussade	306	43.6	MP	Swine/Forestry Workers	France
**J Clin Virol**	**2013**	**Rossi-Tamisier**	**64**	**10.9**	**Adaltis**	**Transplant**	**France**
**J Clin Virol**	**2013**	**Rossi-Tamisier**	**64**	**31.3**	**Wantai**	**Transplant**	**France**
J Infect Dis.	2014	Abravanel	263	38.4	Wantai	Transplant	France
J Med Virol	2008	Mansuy	529	16.6	MP	General Population	France
J Med Virol	2011	Kaba	184	4.4	Adaltis	Immune Deficiency	France
J Virol	2014	Renou	315	7.74	Wantai	General Population	France
Euro Surveill	2015	Mansuy	3353	39	Wantai	General Population	France
Am J Transplant	2012	Pischke	274	11	MP	Transplant	Germany
Emerg Infect Dis	2012	Faber	4352	16.8	Mikrogen	General Population	Germany
**Hepatology**	**2014**	**Wenzel**	**1092**	**14.5**	**Mikrogen**	**General Population**	**Germany**
**Hepatology**	**2014**	**Wenzel**	**1092**	**34**	**Other**	**General Population**	**Germany**
J Clin Microbiol.	2012	Vollmer	200	6	Mikrogen	General Population	Germany
**J Infect Dis.**	**2012**	**Wenzel**	**200**	**18**	**Mikrogen**	**General Population**	**Germany**
**J Infect Dis.**	**2012**	**Wenzel**	**200**	**4.5**	**MP**	**General Population**	**Germany**
**J Infect Dis.**	**2012**	**Wenzel**	**200**	**29.5**	**Other**	**General Population**	**Germany**
J Viral Hepat	2010	Pischke	123	5	Abbott	Immune Deficiency	Germany
Liver Transpl	2010	Pischke	108	1	Abbott	General Population	Germany
Liver Transpl	2010	Pischke	129	3	Abbott	Liver Disease	Germany
Liver Transpl	2010	Pischke	262	4	Abbott	Transplant	Germany

**Med Mibrobiol Immun**	**2014**	**Krumbholz**		**235**	**8.5**	**Mikrogen**	**General Population**	**Germany**
**Med Mibrobiol Immun**	**2014**	**Krumbholz**		**235**	**2.6**	**MP**	**General Population**	**Germany**
**Med Mibrobiol Immun**	**2014**	**Krumbholz**		**235**	**7.7**	**Other**	**General Population**	**Germany**
**Med Mibrobiol Immun**	**2014**	**Krumbholz**		**302**	**17.9**	**Mikrogen**	**Swine/Forestry Workers**	**Germany**
**Med Mibrobiol Immun**	**2014**	**Krumbholz**		**302**	**3.5**	**MP**	**Swine/Forestry Workers**	**Germany**
**Med Mibrobiol Immun**	**2014**	**Krumbholz**		**302**	**13.2**	**Other**	**Swine/Forestry Workers**	**Germany**
Med Microbiol Immunol	2012	Krumbholz		116	15.5	Mikrogen	General Population	Germany
Med Microbiol Immunol	2012	Krumbholz		106	28.3	Mikrogen	Swine/Forestry Workers	Germany
Med Microbiol Immunol	2012	Dremsek		563	11	Mikrogen	General Population	Germany
Med Microbiol Immunol	2012	Dremsek		301	18	Mikrogen	Swine/Forestry Workers	Germany
PLoS One	2014	Pischke		114	3.5	MP	Immune Deficiency	Germany
PLoS One	2014	Pischke	sub-cohort 1	208	7.7	MP	Liver Disease	Germany
PLoS One	2014	Pischke	sub-cohort 2	109	2.8	MP	Liver Disease	Germany
Transfusion	2014	Juhl		1019	6.8	Mikrogen	General Population	Germany
Transpl Infect Dis	2012	Koencke		52	3.8	Other	Transplant	Germany
Transpl Infect Dis	2014	Pischke		537	2	MP	General Population	Germany
Transpl Infect Dis	2014	Pischke		537	2	MP	General Population	Germany
Transpl Infect Dis	2014	Pischke		95	5.3	MP	Transplant	Germany
BMC Infect Dis	2015	Schielke		126	21	Mikrogen	Swine/Forestry Workers	Germany

Epidemiol Infect	2014	Scotto	General Population	450	2.7	Dia.Pro	General Population	Italy
Epidemiol Infect	2014	Scotto	Blood Donors	151	1.3	Dia.Pro	General Population	Italy
Epidemiol Infect	2013	Scotto		100	2	Dia.Pro	Immune Deficiency	Italy
Infection	2013	Rapicetta		973	11.6	Other	Liver Disease	Italy
J Prev Med Hyg	2009	Maisa		130	2.3	Other	General Population	Italy
J Prev Med Hyg	2009	Masia		402	5	Other	Swine/Forestry Workers	Italy
World J Gastroenterol	2015	Scotto		450	2.7	Dia.Pro	General Population	Italy
World J Gastroenterol	2015	Scotto		120	3.3	Dia.Pro	Transplant	Italy
J Clin Virol	2014	Hassing		256	11.7	Wantai	Immune Deficiency	Netherlands
Blood	2013	Versluis		328	13	Dia.Pro	Transplant	Netherlands
Epidemil Infect	2012	Verhoef		7072	1.9	MP	General Population	Netherlands
**Epidemiol Infect**	**2008**	**Bouwknegt**		**644**	**1.7**	**Abbott**	**General Population**	**Netherlands**
**Epidemiol Infect**	**2008**	**Bouwknegt**		**644**	**4.2**	**MP**	**General Population**	**Netherlands**
**Epidemiol Infect**	**2008**	**Bouwknegt**	**swine veterianrians**	**49**	**12.2**	**MP**	**Swine/Forestry Workers**	**Netherlands**
**Epidemiol Infect**	**2008**	**Bouwknegt**	**swine veterianrians**	**49**	**8.1**	**Abbott**	**Swine/Forestry Workers**	**Netherlands**
**Epidemiol Infect**	**2008**	**Bouwknegt**	**non-swine veterinarians**	**153**	**5.2**	**Abbott**	**Swine/Forestry Workers**	**Netherlands**
**Epidemiol Infect**	**2008**	**Bouwknegt**	**non-swine veterinarians**	**153**	**3.9**	**MP**	**Swine/Forestry Workers**	**Netherlands**
Euro Surveill/Transfusio n Hogema same collective	2013	Slot		5239	27	Wantai	General Population	Netherlands
J Viral Hepat	2007	Herremans		50	4.2	MP	General Population	Netherlands
J Viral Hepat	2007	Herremans		1027	2.3	Other	Liver Disease	Netherlands

Liver Transpl	2009	Haagsma		285	2.1	MP	Transplant	Netherlands
Transfusion	2014	Hogema		5329	27	Wantai	General Population	Netherlands
AIDS	2014	Pineda		613	26	Wantai	Immune Deficiency	Spain
Am J Trop Med Hyg	2008	Galiana		101	18.8	Wantai	Swine/Forestry Workers	Spain
Am J Trop Med Hyg	2008	Galiana		97	4.1	Wantai	General Population	Spain
Clin Vaccine Immuno	2006	Buti		1280	7.3	Other	General Population	Spain
Gastroenterol Hepatol	2004	Sutrez Gonztlez		325	0.6	Abbott	General Population	Spain
HIV Med	2012	Jardi		238	9	Other	Immune Deficiency	Spain
J Infect	2014	Rivero-Juarez		894	9.8	Wantai	Immune Deficiency	Spain
J Med Virol	2011	Fogeda		2305	2.17	Dia.Pro	General Population	Spain
J Med Virol	2014	Lindemann		448	10.4	Dia.Pro	Immune Deficiency	Spain
J Med Virol	2010	Lindemann		1040	3.6	Dia.Pro	General Population	Spain
PLoS One	2014	Riveiro-Barciela		200	3.5	MP	General Population	Spain
PLoS One	2014	Riveiro-Barciela		238	9.2	MP	Immune Deficiency	Spain
PLoS One	2014	Riveiro-Barciela	sub-cohort 1	57	17.5	MP	Liver Disease	Spain
PLoS One	2014	Riveiro-Barciela	sub-cohort 2	301	5	MP	Liver Disease	Spain
PLoS One	2014	Riveiro-Barciela	sub-cohort 1	332	9.5	MP	Transplant	Spain
PLoS One	2014	Riveiro-Barciela	sub-cohort 2	296	3.7	MP	Transplant	Spain
**Transfusion**	**2014**	**Sauleda**		**10,000**	**10.72**	**Mikrogen**	**General Population**	**Spain**
**Transfusion**	**2014**	**Sauleda**		**10,000**	**19.96**	**Wantai**	**General Population**	**Spain**
PloS One	2011	Kaufmann		550	4.9	MP	General Population	Switzerland
Emerg Infect Dis	2011	Kenfak-Foguena		735	2.4	MP	Immune Deficiency	Switzerland

**PLoS One**	**2013**	**Schnegg**	**550**	**4.9**	**MP**	**General Population**	**Switzerland**
**PLoS One**	**2013**	**Schnegg**	**550**	**4.2**	**Dia.Pro**	**General Population**	**Switzerland**
**PLoS One**	**2013**	**Schnegg**	**550**	**21.2**	**Wantai**	**General Population**	**Switzerland**
Emerg Infect Dis	2013	Payne	141	3.5	Wantai	General Population	UK
Emerg Infect Dis	2013	Payne	146	7.1	Wantai	Immune Deficiency	UK
Eur J Clin Microbiol Infect Dis	2008	Dalton	500	16	Wantai	General Population	UK
Eur J Gastroenterol Hepatol	2008	Dalton	126	13	MP	Liver Disease	UK
HIV Med	2011	Keane	464	13.8	Wantai	General Population	UK
HIV Med	2011	Keane	138	9.4	Wantai	Immune Deficiency	UK
J Clin Virol.	1991	Ijaz	1591	13.5	Wantai	General Population	UK
J Clin Virol.	2004	Ijaz	1140	13	Wantai	General Population	UK
J Med Virol	2013	Harrions	670	18.8	Wantai	General Population	UK
J Med Virol	2013	Harrions	76	36.8	Wantai	Haemodialysis	UK
J Med Virol	2013	Harrions	88	18.2	Wantai	Transplant	UK
**J Med Virol**	**2010**	**Bendall**	**500**	**3.6**	**MP**	**General Population**	**UK**
**J Med Virol**	**2010**	**Bendall**	**500**	**16.2**	**Wantai**	**General Population**	**UK**
Vox Sang	2013	Cleland	1559	4.7	Wantai	General Population	UK
Zoonoses Public Health	2010	Meader	412	2.4	MP	General Population	UK

© 2016 by the authors; licensee MDPI, Basel, Switzerland. This article is an open access article distributed under the terms and conditions of the Creative Commons Attribution (CC-BY) license (http://creativecommons.org/licenses/by/4.0/).
